# Psychoactive substances use and associated factors among middle and high school students in the North Center of Morocco: a cross-sectional questionnaire survey

**DOI:** 10.1186/s12889-016-3143-5

**Published:** 2016-06-04

**Authors:** B. Zarrouq, B. Bendaou, A. El Asri, S. Achour, I. Rammouz, R. Aalouane, B. Lyoussi, S. Khelafa, A. Bout, N. Berhili, H. Hlal, A. Najdi, C. Nejjari, K. El Rhazi

**Affiliations:** Laboratory of Epidemiology, Clinical Research, and Health Community, Faculty of Medicine and Pharmacy, Sidi Mohammed Ben Abdallah University, Fez, Morocco; Laboratory of Physiology-Pharmacology and Environmental Health, Dhar El Mahraz Faculty of Sciences, Sidi Mohammed Ben Abdallah University, Fez, Morocco; Unit of Toxicology, Hassan the 2nd University Hospital Center, Fez, Morocco; Laboratory of Clinical Neurosciences, Faculty of Medicine and Pharmacy, Sidi Mohammed Ben Abdallah University, Fez, Morocco; Service of Psychiatry, Ibn Al Hassan Hospital, Hassan the 2nd University Hospital Center, Faculty of Medicine and Pharmacy, Sidi Mohammed Ben Abdallah University, Fez, Morocco

**Keywords:** Prevalence, Psychoactive substance, Risk factors, Secondary school, School students, Morocco

## Abstract

**Background:**

Data on psychoactive substance (PAS) consumption among adolescents in the North Center of Morocco are not at all available. Therefore, the current study aimed at investigating the prevalence and the determinants of psychoactive substances use among middle and high school students in this region.

**Methods:**

A cross-sectional study was conducted from April 2012 to November 2013 in public middle and high schools in the North Central Region of Morocco. An anonymous self-administered questionnaire was used to assess psychoactive substances use among a representative sample of school students from the 7th to the 12th grade, aged 11–23 years, selected by stratified cluster random sampling. Factors associated with psychoactive substance use were identified using multivariate stepwise logistic regression analyses.

**Results:**

A total of 3020 school students completed the questionnaires, 53.0 % of which were males. The overall lifetime smoking prevalence was 16.1 %. The lifetime, annual and past month rates of any psychoactive substance use among the study subjects were 9.3, 7.5, and 6.3 % respectively. Cannabis recorded the highest lifetime prevalence of 8.1 %, followed by alcohol 4.3 %, inhalants 1.7 %, psychotropic substances without medical prescription 1.0, cocaine 0.7, heroine 0.3, and amphetamine with only 0.2 %. Psychoactive substance use was associated with males more than females. The risk factors identified by multivariate stepwise logistic regression analyses were being male, studying in secondary school level, smoking tobacco, living with a family member who uses tobacco, and feeling insecure within the family.

**Conclusions:**

The prevalence among all school students reported by the current study was comparable to the national prevalence. Efforts to initiate psychoactive substance prevention programs among school students should be made by designing such programs based on the significant factors associated with psychoactive substance use identified in this study.

**Electronic supplementary material:**

The online version of this article (doi:10.1186/s12889-016-3143-5) contains supplementary material, which is available to authorized users.

## Background

Globally, it was estimated that a total of 246 million people, or 1 out of 20 people between the ages of 15 and 64 years, had used an illicit drug in 2013. The magnitude of the world drug problem becomes more apparent when considering that more than 1 out of 10 drug users is a problem drug user, suffering from drug use disorders or drug dependence [1].

In developing countries, recent trends indicate that the use of psychoactive substances (PASs) have dramatically increased [2]. In Morocco, limited information is available on the epidemiology of substance use. The national survey conducted among the general population on mental disorders and addiction in 2003 and 2006 found that the prevalence of PASs consumption for people aged more than 15 was 4.1 % [3]. According to the annual report of the National Observatory of drugs and addictions, cannabis was the most common illegal drug used by Moroccan adolescents in 2013 [4].

In fact, Morocco is one of the main producers of cannabis resin (hashish) in the world despite the intensified efforts of the authorities to decrease the area under cultivation (134,000 ha in 2003 to 47,196 ha in 2013) [1, 5]. The Rif Mountains of the Northern Region is the main area of cannabis cultivation in Morocco [6]. According to the Morocco cannabis survey (2005), 86.0 % of cannabis cultivation was concentrated in three Northern provinces namely Chefchaouen (50.0), Taounate (19.0) and Al Hoceima (17.0 %) [7].

Albeit the development of the cannabis economy in the Northern provinces of Morocco, no study has been conducted to determine the prevalence and the risk factors related to cannabis and other PASs use among adolescents in this region. Therefore, we conducted a cross sectional survey to estimate the prevalence and the determinants of cannabis and PASs use among middle and high school students in the North Center of Morocco.

## Methods

### Study design

A cross-sectional study, targeting students in public middle and high schools of the North central region of Morocco (Fez-Boulemane and Taza Taunate El Hoceima regions), was conducted from April 2012 to November 2013.

### Sampling method

The national survey conducted in 2006 estimated that the lifetime prevalence of PASs use among Moroccan school students was 6.0 % [8]. Henceforth, we calculated the minimum sample size to estimate the prevalence for 95 % confidence limits at an allowable error of 10 %. For sample size estimation under cluster sampling, we considered a value of 2 for design effect. The main components of the design effect are the intraclass correlation, and the cluster sample sizes. In practice, when the cluster sample sizes are between 20 to 50, the intraclass correlation is kept very small. The design effect values available from published reports (e.g., Demographic and Health Survey reports) reported that usually a value of 2 for design effect is considered for sample size estimation under cluster sampling [9]. As a result, we came up with a minimum sample size of 2860 participants.

The list of schools in the North central region was obtained from the Statistical Office of the Ministry of National Education. The schools were split into seven geographical regions and classified into two strata: urban and rural. Forty four middle schools and twenty four high schools were randomly selected. The clusters were defined as classes in each selected school, and all the students of the clusters were involved in the survey. The total cluster selection was done in proportion with the distribution of the schools in urban and rural areas and according to the distribution of students in middle and high schools. This study was conducted according to the guidelines of the Helsinki Declaration.

### Data collection

A questionnaire was developed by reviewing the relevant literature (Additional file 1). The questions were divided into three sections: (i) a socio-demographic background; (ii) smoking tobacco; (iii) and alcohol and PASs use. All participants were asked about their use of tobacco, alcohol, cannabis, psychotropic substances without medical prescription, cocaine, heroin, amphetamine and inhalants (glue, solvents, and aerosols).

Tobacco use was defined according to the International Union against Tuberculosis and Lung Diseases Guide [10]. Lifetime PASs use was also defined as having ever used at least one of the PASs listed in the questionnaire. Past twelve months PASs use was defined as having used the PASs listed in the questionnaires during the 12 months prior to the date of data collection. The current prevalence of PASs use was defined as the proportion of students who used PASs in 30 days preceding the study. At last, Poly-substance use was defined as the regular use of at least two PASs listed in the questionnaire.

### Procedures

The participation in the presented study was entirely voluntary and full confidentiality of the responses was maintained after a clear explanation of the objectives. The absence of any school personnel or teacher at the time of data collection was ensured inside the classrooms to prevent any response bias.

After explaining the instructions for questionnaires’ completion, the five investigators who were resident doctors in psychiatry encouraged participants to work individually, quietly, honestly, and as quickly as they could. The time given for participants to fill the questionnaire was from 30 min to an hour.

### Data quality control and analysis

The data entry stage started immediately after finishing data collection. The data were entered into MS Windows Excel in the form of codes, and transferred to Statistical Package for Social Sciences SPSS version 20 (SPSS Inc., Chicago, IL, USA). Data analysis involved descriptive statistics as well as univariate and multivariate analysis. Descriptive statistics involved frequencies, and proportion for categorical variables including cross tabulations. Univariate associations between PAS use and sociodemographic variables and tobacco use were assessed using the chi-square test. Variables with *P* ≤ 0.20 on univariate analysis were entered in multivariate stepwise logistic regression model to investigate the risk factors of PAS use. The Hosmer-Lemeshow goodness-of-fit test was used to evaluate multivariate model fit (higher p values indicate better fit of the model). *P* values of <0.05 were considered as statistically significant.

## Results

### Socio-demographic characteristics

Out of the 3170 students who participated in the study, 3020 completed the questionnaires (at a response rate of 95.0 %). About 54.9 % of participants were from middle school; while, 45.1 % were from high school. More males (53.0 %) than females (47.0 %) participated in this study, and the average age was 16 years (SD ± 2 years). As shown in Table 1, 24.7 of surveyed students lived in a rural environment and 75.3 % in urban areas. Regarding parent’s marital status, the majority of parents were married (89.3 %). Concerning parents’ education, nearly 35.9 of participants’ fathers and 60.6 % of mothers were illiterate; only 11.0 % of fathers and 5.0 % of mothers held university degrees. Regarding family income, most students (76.9 %) were from medium income families; 88.4 % of students’ fathers were employed whereas, 90.5 % of students’ mothers were unemployed.

**Table 1:** Some selected background characteristics of the study population (*n* = 3020)

Characteristics	*N*	%
Gender		
Boys	1602	53.0
Girls	1418	47.0
Age groups (years)		
11–14	795	26.4
15–18	1857	61.7
19–23	357	11.9
School levels		
Middle school	1657	54.9
High school	1363	45.1
Location of school		
Urban	2273	75.3
Rural	774	24.7
Parents’ marital status		
Married	2684	89.3
Divorced	109	3.7
Widowed	145	4.9
Separated	65	2.1
Father’s education		
Illiterate	1065	35.9
Primary	806	27.2
Secondary	767	25.9
University and above	326	11.0
Mother’s education		
Illiterate	1813	60.6
Primeray	514	17.2
Secondary	517	17.3
University and above	149	5.0
Father employment		
Employed	2490	88.4
unemployed	82	2.9
Retired	245	8.7
Mother employment		
Employed	281	9.5
unemployed	2688	90.5
Family income		
≤ 300 £	498	16.8
301 – 1000 £	2280	76.9
≥ 1000 £	187	6.3

### Tobacco use

Overall, the lifetime smoking prevalence among all school students was 16.1 % (95 % CI: 14.8 % - 17.4 %) (Table 2). The prevalence of current smokers was 9.1 % (95 % CI: 8.1 % - 10.2 %), with mean consumption of 6.5 cigarettes (SD = 6.1) per day. Male students were more likely to current smoke than females (15.0 % vs. 2.5 %, *p* < 0.001). The mean age at which the school students started smoking cigarettes was 13.9 years (SD ± 2.5 years).

**Table 2:** Prevalence of tobacco and psychoactive substance use among middle and high school students by gender

Tobacco and psychoactive substance use	Male (*n* = 1588)		Female (*n* = 1405)		Total (*n* = 2993)	
	*N*	%	*N*	%	*N*	%
Tobacco						
Lifetime	416	26.2	69	4.9	485	16.1
Last 12 months	240	15.0	35	2.5	275	9.1
Last 30 days	159	10.0	17	1.2	176	5.9
Psychoactive substances						
Lifetime	250	15.7	30	2.1	280	9.3
Last 12 months	207	13.0	17	1.2	224	7.5
Last 30 days	175	11.0	13	0.9	188	6.3
Cannabis						
Lifetime	215	13.5	27	1.9	242	8.1
Last 12 months	185	11.6	16	1.1	201	6.7
Last 30 days	156	9.8	13	0.9	169	5.6
Alcohol						
Lifetime	119	7.5	10	0.7	129	4.3
Last 12 months	105	6.6	6	0.4	111	3.7
Last 30 days	93	5.8	4	0.2	97	3.2
Inhalants						
Lifetime	48	3.0	2	0.2	50	1.7
Last 12 months	45	2.8	1	0.1	46	1.5
Last 30 days	35	2.2	1	0.1	36	1.2
Psychotropic substances without medical prescription						
Lifetime	29	1.8	1	0.1	30	1.0
Last 12 months	27	1.7	1	0.1	28	0.9
Last 30 days	20	1.2	1	0.1	21	0.7
Cocaine						
Lifetime	18	1.1	2	0.1	20	0.7
Last 12 months	18	1.1	2	0.1	20	0.7
Last 30 days	11	0.7	0	0.0	11	0.4
Heroine						
Lifetime	7	0.4	1	0.1	8	0.3
Last 12 months	6	0.3	1	0.1	7	0.2
Last 30 days	3	0.2	0	0.0	3	0.1
Amphetamine						
Lifetime	5	0.3	0	0.0	5	0.2
Last 12 months	5	0.3	0	0.0	5	0.2
Last 30 days	2	0.1	0	0.0	2	0.1

### Psychoactive substances use

The overall lifetime prevalence of any PAS use among the study subjects was 9.3 % (95 % CI: 8.4 % – 10.5 %). Males were more likely than females to use PASs (15.7 % vs. 2.1 %, *p* < 0.001). The last 12 months and the last 30 days prevalence of PASs use among school students were 7.5 and 6.3 % respectively.

Table 2 provides the lifetime, the last 12 months, and the current prevalence of PAS use among males and females participants. Cannabis recorded the highest lifetime prevalence with 8.1 %, followed by alcohol (4.3 %), inhalants (1.7 %), psychotropic substances without medical prescription (1.0), cocaine (0.7), heroine (0.3), and amphetamine with only 0.2 %. In general, the rate of poly drug use (two or more PASs) was 30.0 %.

The mean age at which the respondents started using PASs was 14.6 years (SD ± 2.5 years). The majority of the school students (37.1 %) started using PASs out of curiosity; while, 34.4 % consumed it to reach certain degree of happiness or pleasure; indeed, 27.7 % admitted using PASs to overcome family problems; 23.0 % considered it helpful to get over personal problems; whereas, 19.1 % stated that they did it because of peer pressure.

### Univariate and multivariate analysis

As shown in Table 3, statistically significant associations (*P* < 0.05) were observed between the PASs status, age groups, school levels, and family income. The PASs use prevalence was higher among high school students (13.4 %), especially in participants aged 19–23 years (22.4 %), and proved lower among students from medium income families (8.4 %)*.* No significant associations were observed in relation to the students’ locations (urban or rural areas), parents’ marital status, fathers or mothers education, and fathers or mothers employment.

**Table 3:** Psychoactive substance use and background characteristics

	Users						Non-users						*p-*value
Background characteristics	Male		Female		Total		Male		Female		Total		
	*N*	%	*N*	%	*N*	%	*N*	%	*N*	%	*N*	%	
School levels													
Intermediate	98	10.5	3	0,4	101	6.1	836	89.5	720	99.6	1556	93.9	<0.001
Secondary	152	23.2	27	3,9	179	13.4	502	76.7	655	96.0	1157	86.6	
Age groups (years)													
11–14	13	3.2	1	0,3	14	1.8	387	96.8	394	99.7	781	98.2	<0.001
15–18	164	17.1	23	2.6	187	10.1	796	82.9	865	97.4	1661	89.9	
19–23	70	31.5	6	5.1	76	22.4	152	68.5	111	94.9	263	77.6	
Location													
Urban	190	16.9	28	2.5	218	9.7	935	83.1	1094	97.5	2029	90.3	NS
Rural	60	12.9	2	0.7	62	8.3	403	87.1	281	99,3	684	91.7	
Parents’ marital status													
Married	219	15.4	22	1.8	241	9.1	1201	84.6	1218	98.2	2419	90.9	NS
Divorced	9	16.9	4	7.1	13	11.9	44	83.1	52	92.9	96	88.1	
Widowed	15	20.5	2	2.9	17	11.9	58	79.5	68	97.1	126	88.1	
Separated	4	14.3	1	2.7	5	7.7	24	85.7	36	97.3	60	92.3	
Father’s education													
Illiterate	77	13.1	2	0.5	79	7.5	514	86.9	465	99.5	979	92.5	NS
Primary	72	16.9	11	3.0	83	10.5	353	83.1	357	97.0	710	89.5	
Secondary	71	19.1	10	2.5	81	10.6	301	80.9	381	97.5	682	89.4	
University and above	23	13.2	6	4.0	29	9.0	151	86.8	144	96.0	295	91.0	
Mother’s education													
Illiterate	165	16.8	8	1.0	173	9.6	813	83.2	807	99.0	1620	90.4	NS
Primeray	29	11.2	7	2.8	36	7.1	231	88.8	242	97.2	473	92.9	
Secondary	37	14.3	10	3.9	47	9.1	221	85.7	248	96.1	469	90.9	
University and above	13	17.1	4	5.5	17	11.4	63	82.9	69	94.5	132	88.6	
Father employment													
Employed	204	15.2	26	2.3	230	9.3	1136	84.8	1101	97.7	2273	90.7	NS
unemployed	8	18.2	0	0.0	8	9.9	36	81.8	37	100.0	73	90.1	
Retired	19	17.2	1	0.8	20	8.2	91	82.8	133	99.2	224	90.8	
Mother employment													
Employed	18	12.8	9	6.4	27	9.6	123	87.2	131	93.6	254	90.4	NS
unemployed	218	15.4	19	1.5	237	8.9	1195	84.6	1231	98.5	2426	91.1	
Family income													
≤ 300 £	58	20.5	4	1.9	62	12.6	225	79.5	205	98.1	430	87.4	<0.007
301 – 1000 £	165	14.3	24	2.2	189	8.4	990	85.7	1081	97.8	2071	91.6	
≥ 1000 £	21	18.3	1	1.4	22	11.8	94	81.7	71	98.6	165	88.2	

*NS* Not significant

*p* < 0.05

Potential risk factors of PASs use were displayed in Table 4. These factors involved gender, school level, age group, smoking tobacco status, friends and family member smoking status, and feelings of insecurity within family. Univariate analyses revealed that all of the previously stated factors exhibited highly significant statistical associations (*P <*0 .002), with odd ratios ranging from 1.9 (95 % CI: 1.3–3.0) for friends who smoke to 46.6 (95 % CI: 33.4– 65.2) for smoking tobacco status.

**Table 4:** Univariate and multivariate logistic regression analysis for psychoactive substance use related factors among participants

	Univariate^a^			Multivariate^b^		
Category	OR	95 % CI	*p*- value	OR	95 % CI	*p*- value
Gender						
Female	1			1		
Male	8.5	5.8–12.6	<0.001	1.9	1.1–2.7	<0.05
School levels						
Intermediate	1			1		
Secondary	2.3	1.8–3.1	<0.001	2.8	1.6–4.8	<0.001
Age groups (years)						
11–14	1			1		
15–18	2.5	1.9–3.4	<0.001	1.0	0.3–3.4	NS
19–23	16.1	8.9–29.4	<0.001	1.1	0.6–2.1	NS
Feel secure						
Yes	1			1		
No	2.1	1.6–2.8	<0.001	2.1	1.1–3.9	<0.02
Smoking tobacco						
No	1			1		
Yes	46.6	33.4–65.2	<0.001	27.3	19.2–39.0	<0.001
Family member who smoke						
No	1			1		
Yes	2.3	1.3–3.9	<0.002	2.9	1.6–5.5	<0.001
Friends who smoke						
No	1			1		
Yes	1.9	1.3–3.0	<0.002	1.9	1.2–3.1	<0.005

*OR* Odds ratio

95 % CI 95 % confidence intervals

*p* < 0.05

^a^Univariate associations between PAS use and sociodemographic variables and tobacco use were assessed using the chi-square test

^b^Variables with *p* ≤ 0.20 on univariate analysis were entered in multivariate stepwise logistic regression model

The Hosmer-Lemeshow goodness-of-fit test was used to evaluate multivariate model fit (χ2 = 2.2, *p* = 0.9)

In multivariate logistic regression analysis, age group exhibited statistically non-significant association. The risk factors identified by the multivariate stepwise logistic regression analysis were being male (OR: 1.9; 95 % CI: 1.1 – 2.7), studying in secondary school (OR: 2.8; 95 % CI: 1.6 – 4.8), smoking tobacco (OR: 27.3; 95 % CI: 19.2 – 39.0), having a family member who smokes (OR: 2.9; 95 % CI: 1.6 – 5.5), having a friend who uses tobacco (OR: 1.9; 95 % CI: 1.2 – 3.1), and feeling insecure within the family (OR: 2.1; 95 % CI: 1.1 – 3.8). The Hosmer- Lemeshow goodness-of-fit test was not significant (χ^2^ = 2.2, *p* = 0.9), indicating the model fit the data well.

## Discussion

### Tobacco use

The present study is the first large survey focusing on PAS use among middle and high school students in the North Center of Morocco.

The lifetime smoking prevalence among all school students (16.1 %) reported in this study was slightly lower than the rate (17.0 %) reported by Mediterranean School Survey Project on Alcohol and Other Drugs (MedSPAD), which is a cross disciplinary survey that was conducted in Moroccan schools in 2013 among 5801 school students [11]. Considering only the results for subjects aged 13–15 years, the prevalence of current tobacco use was 4.0 %. This rate was lower than the findings reported by the Global School-based Student Health Survey (GSHS) conducted in Morocco and other nearby countries among students aged 13–15 years. The 2010 Morocco GSHS, which was conducted among 2924 students, indicated that the prevalence of current smoking cigarettes was 5.2 % [12]. In Tunisia, 7.5 % of students who participated in the 2008 GSHS, reported the use of tobacco one or more days during the past 30 days [13]. In Algeria, the 2011 GSHS, which was conducted among 4532 school students, estimated that the prevalence was 9.2 % [14]. The 2010 Mauritania GSHS revealed that 17.3 % of school students were currently tobacco users [15]. Low tobacco use rate among school students in the North Center of Morocco could be related mostly to the social and family values in conservative societies, which frown upon tobacco use especially among adolescents.

### Cannabis use

Cannabis recorded the highest lifetime prevalence among illicit drugs (8.1 %); its prevalence in the last 12 months and in the last 30 days was 6.7, and 5.6 %, respectively. These rates were very close to those reported by the national survey 2013 MedSPAD (the life time, the last 12 months, and the last 30 days prevalence were respectively, 9.0, 6.0 and 6.0 %) [11]. Regarding school students aged 13–15 years, the life time prevalence of cannabis (2.8 %) was almost similar to 2010 Morocco GSHS (3.0 %) [12].

In our sample, cannabis is the most-used substance after tobacco. Despite its legal prohibition, cannabis is widely used among Moroccan youth. The availability and accessibility of cannabis can be explained by the fact that Morocco is one of the main producers of cannabis resin in the world. Nevertheless, it appears that cannabis use rates in Morocco are much lower than the rates found among European and American youth. In Europe, 14.6 million or 11.2 % of young adults (15–34) used cannabis in the last year [16]. In North America, annual prevalence of cannabis use is approximately 10.7 % in population aged 15–64, and has increased among youth over the past four years [17].

### Alcohol use

After tobacco and cannabis, the most commonly used substance in our sample is alcohol. These findings were consistent with what was reported by the National Observatory of drugs and addictions in 2014 [4]. Still, the lifetime, annual and past month rates (4.3, 3.7, and 3.2 % respectively) announced by the present study, were lower than national prevalence (8.0, 5.0, and 5.0 % respectively) [11]. The comparison with other Arab countries was difficult because recently measured prevalence rates are not always available. Nevertheless, it appears that alcohol use among Moroccan school students falls in middle grounds in relation to other neighboring countries. In Tunisia, the national survey conducted in 2008 among middle school students revealed that 3.8 % of adolescents used alcohol and other drugs once or more times during their life [13]. The past month prevalence of alcohol among school students in Syria and Lebanon in 2010/11 was 7.4 and 28.5 %, respectively [18, 19].

According to the world health organization (WHO), the lowest prevalence rates of heavy episodic drinking among adolescents aged 15–19 in the world for 2010, were recorded in Muslim countries located in the Eastern Mediterranean Region, such as Morocco [20], which can be relegated to alcohol religious prohibition.

### Other psychoactive substances use

The present study showed that the lifetime prevalence of inhalants, psychotropic substances without medical prescription, cocaine, heroin and amphetamine use were 1.7, 1.0,0.7, 0.3, and 0.2 % respectively. Comparing the prevalence of inhalants and amphetamine use with other regions of Morocco was not possible because of the dearth of research on the use of these substances among Moroccan school students.

On the other hand, the lifetime prevalence for cocaine and heroin use reported by this study was lower than the rate estimated by the 2013 national survey (cocaine 1.3 %, heroine 1.2 %) [11]. Generally, the prevalence of cocaine and heroin use among Moroccan school students remain lower than the rates found in some Arab countries like Lebanon, and much lower than those reported among European youth [11, 16]. The plausible reason for this low prevalence might be related to financial and accessibility considerations. As a matter of fact, Moroccan students don’t have enough money, or easy access to cocaine and heroin. Contrariwise, the most used substances by Moroccans students (tobacco, cannabis and alcohol) were the most available and the most accessible PASs.

For the consumption of psychotropic substances without medical prescription, the rate found in this study (1.0 %) was lower than the national lifetime prevalence (5.0 %) [11]. Moreover, our results did not show gender differences concerning the preference of psychotropic substances without medical prescription conversely to the findings of five recently surveyed countries (Australia, United States of America, Spain, Urban Afghanistan, and Pakistan), which reported that the non-medical use of pharmaceutical drugs was nearly equivalent, not to say higher among women [21, 22]. Likewise, the national survey conducted in Morocco in 2009 indicated that the prevalence of tranquillizers or sedatives use without doctors’ prescriptions exceeded the rate of cannabis use among Moroccan females aged 15–17 [23]. Similarly, in Algeria, there was an evident preference for psychotropic drugs among females 15–16 years old, which did not only exceed cannabis use but also alcohol and tobacco use as well [24]. The fact that psychotropic drugs were first in use among girls might suggest that social acceptance and accessibility of such drugs (a prescription issued by an obliging doctor) fostered this consumption preference among girls.

### Risk factors of psychoactive substances use

Our results demonstrated that the potential risk factors of PASs use included gender, school levels, age groups, smoking tobacco status, friends and family member smoking status, and feelings of insecurity within family.

In the present study, males were more likely to use PASs than females. For lifetime, annual and past-month prevalence of the use of the entire studied PASs, the rates reported among males were significantly higher than females. These findings confirmed national findings that there was a predominance of PASs use among boys [11], and international findings that nearly all drug use surveys indicated that male–female differences in the use of alcohol and drugs occurred in most countries, with males being more likely than females to use at all [21, 25–27]. However, it should be noted that in Morocco, the use of PASs among females has been associated with social stigmatization. Smoking or using drugs is still seen as shameful and inappropriate. As a result, females are not likely to report their use of PAS honestly, and this is probably the source of bias which could drastically underestimate the prevalence of PAS use among females*.*

In terms of other determinants of PAS use, we observed that the prevalence of PASs use was correlated positively with age. This result was consistent with what was often reported in developing countries [28]. For the mean age of onset of PAS, we found that the average was 14 years, which means that young people initiate PASs use during late middle school and early high school [29]. Early initiation of substances can lead to the host of current and future substance use problems such as poor school performance, school drop-out or delinquency, health problems, and future risks for substance use disorders [30, 31].

Results of the present study indicated that students consumption of PASs was related to smoking status; current tobacco users were at higher risk of PASs use than non-users (OR = 27.3). In fact, the temporal sequencing of PASs use, as it was observed across several epidemiological samples, is cigarettes and alcohol initiation, followed by cannabis, and finally other PASs. Such a finding points to the pivotal role of early initiation of cigarette or alcohol use on subsequent PASs use [32, 33].

This study indicated that PAS use by friends and family member, alongside with feelings of insecurity within the family were significant risk factors (OR_1_ = 1.9, OR_2_ = 2.9, and OR_3_ = 2.1, respectively). These results have been congruent with those of previous studies that have showed that social factors are important determinants of the risk of PASs experimentation, regular use, and addiction in adolescence. Risk factors such as family dysfunction, bad relationship with parents, having family member who used PASs, or being friends with smokers or users of PASs are more decisive in the overall intensity of PASs use in males than in females [34].

We note some limitations to this study. First, PASs use was self-reported, which could be subject to social desirability bias. Since PASs use is a taboo practice in the Moroccan conservative society, and constitutes a felony under the Moroccan law, there is a risk of underreporting among school students generally and females particularly when using self-report questionnaires. Although we went to great lengths to ensure confidentiality and anonymity, we could not assess underreporting of substance use. The prevalence reported above may thus represent low estimates of the actual prevalence. Second, the study was cross-sectional, which limits the extent to which conclusions can be drawn about the causal nature of the associations between the correlates and PASs use.

Concerning the strengths of the present study, it includes a large representative sample. It is the first study of its kind to be performed among middle and high school students in the North Center of Morocco with the aim of investigating the prevalence and exploring the risk factors of PASs use.

## Conclusion

Our data maintain the idea that many students initiate PASs consumption during late middle school and early high school. As vindicated throughout our findings, current tobacco users were at higher risk of PASs use than non-users. The presented results call attention to the urgent need of implementing prevention strategies at an early age. Hence, the first step to counteract the PASs abuse is to spread awareness against tobacco consumption. Monitoring PASs use among Moroccan school students is essential to study risky behaviors among adolescents and provide a pointer to potential future trends.

## Abbreviations

GSHS, Global school health survey; MedSPAD, Mediterranean School Survey Project on Alcohol and Other Drugs; PASs, Psychoactive substances

## Additional file

Additional file 1: English version of the questionnaire. (DOCX 27 kb)
