# Supplementary material for: Psychoactive substances use and associated factors among middle and high school students in the North Center of Morocco: a cross-sectional questionnaire survey
Source: BMC Public Health. 2016 Jun 4;16:468. doi: 10.1186/s12889-016-3143-5 (PMC4893420; doi:10.1186/s12889-016-3143-5)
Supplement: Additional file 1: — English version of the questionnaire. (DOCX 27 kb) [file 12889_2016_3143_MOESM1_ESM.docx]

Questionnaire ID……………

**Sidi Mohammed Ben Abdallah University**

**Dhar El Mahraz Faculty of Sciences**

**Ibn Al Hassan Hospital**

**Hassan the 2^nd^ University Hospital Center**

**Faculty of Medicine and Pharmacy, Fez, Morocco**

***A Questionnaire on Psychoactive Substances Use***

| **Read this first please!**  Dear Student,  This questionnaire is a part of a study conducted on psychoactive substances use among Moroccan school students. It will be answered by more than 3000 students in the regions of Fez, Boulemane, Taza, Tawnat, and Hoceima.  Please, do not write your name on this questionnaire. Hence, nobody will be able to identify who has completed this particular form.  Please, answer as thoughtfully and as honestly as possible all the questions. This is not a test; therefore, there are no ‘right’ or ‘wrong’ answers.  Your class has been randomly selected to take part in this study. Completing the survey is voluntary. Your grade or mark in this class will not be affected whether or not you answer the questions. If you do not want to answer a particular question, just leave it blank.  Please, mark the appropriate answer to each question by making an "X" in the box. If you have a question, please raise your hand and your survey investigator will assist you.  **Thank you in advance for your participation! Please begin.** |
| --- |

**THE NEXT TEN QUESTIONS ASK FOR SOME BACKGROUND INFORMATION ABOUT YOURSELF**

1. What is your sex?

❒ Male

❒ Female

1. How old are you? …….. Years
2. In what school level are you?

❒ Middle school

❒ High school

1. What is your school district?

❒ Urban

❒ Rural

1. As of today, what is the marital status of your legal Parents?

❒ Married

❒ Divorced

❒ Widowed

❒ Separated

#### What is your father’s highest completed level of education?

❒ No formal education

❒ Primary (1-6)

❒ Secondary (7-12)

❒ University and above

❒ I don’t know

1. What is your mother’s highest completed level of education?

❒ No formal education

❒ Primary (1-6)

❒ Secondary (7-12)

❒ University and above

❒ I don’t know

1. What does your father do?

❒ Employed

❒ Unemployed

❒ Retired

1. What does your mother do?

❒ Employed

❒ Unemployed

❒ Retired

1. What is your family monthly income?

❒ ≤ 3000 DH (300 £)

❒ 3100 – 10000 DH (301 – 1000 £)

❒ ≥ 10000 DH (1000 £)

**THE NEXT EIGHT QUESTIONS ASK ABOUT THE USE OF CIGARETTES FOR YOU AND YOUR ENVIRONMENT**

11- Have you ever tried cigarette smoking, even one or two puffs?

❒ Yes

❒ No

1. Have you smoked cigarette for the last 12 months?

❒ Yes

❒ No

1. Have you smoked cigarette for the last 30 days?

❒ Yes

❒ No

1. How many cigarettes have you smoked during your lifetime?

❒ 0 Cigarettes

❒ Less than 100 cigarettes

❒ More than 100 cigarettes

1. During the past year, have you ever tried to stop smoking cigarettes?

❒ I have not smoked cigarettes during the past year

❒ Yes, I have tried to stop smoking during the past year

❒ No, I have not tried to stop smoking during the past year

1. How long ago did you stop smoking?

❒ I have never smoked cigarettes

❒ I have not stopped smoking

❒ I have stopped smoking 1-3 months ago

❒ I have stopped smoking 4-11 months ago or longer

1. Do you have a family member(s) who smokes?

❒ Yes

❒ No

If you stated Yes, say which member(s) of family…………………………………………

1. Do you have friends who smoke?

❒ Yes

❒ No

**THE NEXT QUESTION ASK ABOUT YOUR USE OF PSYCHOACTIVES SUBSTANCES**

1. **Have you ever used psychoactive substances, if you stated “YES”, please fill in the table?**

❒ Yes

❒ No

| Psychoactive substances | Lifetime use | Last 12 months use | Last 30 days use |
| --- | --- | --- | --- |
| Cannabis |  |  |  |
| Alcohol  (beer, wine, spirits) |  |  |  |
| Inhalants (glue, solvents, and aerosols) |  |  |  |
| Psychotropic substances without medical prescription |  |  |  |
| Cocaine |  |  |  |
| Heroine |  |  |  |
| Amphetamine |  |  |  |

**THE NEXT TWO QUESTIONS ASK ABOUT THE MOTIVES BEHIND PSYCHOACTIVES SUBSTANCES USE**

1. Why did you start using psychoactive substances?

❒ Out of curiosity

❒ Due to peer pressure

❒ To reach happiness or pleasure

❒ To overcome family problems

❒ To overcome personal problems

1. Do you feel secure within your family?

❒ Yes

❒ No

**Thank you for your collaboration**
